# Characterization of neoplastic cells outlining the cystic space of invasive micropapillary carcinoma of the canine mammary gland

**DOI:** 10.1186/s12917-021-02807-y

**Published:** 2021-03-24

**Authors:** Michele A. Rodrigues, Andre L. Caldeira-Brant, Dawidson A. Gomes, Tatiany L. Silveira, Hélio Chiarini-Garcia, Geovanni D. Cassali

**Affiliations:** 1 Department of General Pathology, Universidade Federal de Minas Gerais, Av. Antônio Carlos 6627, Belo Horizonte, Minas Gerais CEP: 31270-901 Brazil; 2 Department of Morphology, Universidade Federal de Minas Gerais, Av. Antônio Carlos 6627, Belo Horizonte, Minas Gerais CEP: 31270-901 Brazil; 3 Department of Biochemistry and Immunology, Universidade Federal de Minas Gerais, Av. Antônio Carlos 6627, Belo Horizonte, Minas Gerais CEP: 31270-901 Brazil

**Keywords:** Invasive micropapillary carcinoma, Canine, Mammary gland, Cancer, Tumor microenvironment

## Abstract

**Background:**

Invasive micropapillary carcinoma (IMPC) is a rare malignant breast tumor and a variant form of invasive ductal carcinoma that is an aggressive neoplasm of the human breast and canine mammary gland. The importance of the tumor microenvironment in cancer development has gradually been recognized, but little is known about the cell types outlining the cystic space of canine IMPC. This study aimed to characterize the neoplastic cells outlining the cystic space of IMPC.

**Results:**

Immunohistochemistry (IHC), immunofluorescence (IF), superresolution and transmission electron microscopy (TEM) were used to assess the cell types in the cystic areas of IMPCs. Cells expressing the mesenchymal markers alpha-smooth muscle actin (αSMA), Vimentin, and S100A4 outlined the cystic space of IMPC. Furthermore, loss of epithelial cell polarity in IMPC was shown by the localization of MUC1 at the stroma-facing surface. This protein modulates lumen formation and inhibits the cell-stroma interaction. Immunohistochemical and IF staining for the myoepithelial cell marker p63 were negative in IMPC samples. Furthermore, associated with peculiar morphology, such as thin cytoplasmic extensions outlining cystic spaces, was observed under TEM. These observations suggested cells with characteristics of myoepithelial-like cells.

**Conclusions:**

The cells outlining the cystic space of IMPC in the canine mammary gland were characterized using IHC, IF and TEM. The presence of cells expressing αSMA, Vimentin, and S100A4 in the IMPC stroma suggested a role for tumor-associated fibroblasts in the IMPC microenvironment. The reversal of cell polarity revealed by the limited basal localization of MUC1 may be an important factor contributing to the invasiveness of IMPC. For the first time, the cystic space of canine mammary gland IMPC was shown to be delimited by myoepithelial-like cells that had lost p63 expression. These findings may enhance our understanding of the cellular microenvironment of invasive tumors to improve cancer diagnosis and treatment.

**Supplementary Information:**

The online version contains supplementary material available at 10.1186/s12917-021-02807-y.

## Background

One of the most aggressive types of breast cancer is invasive micropapillary carcinoma (IMPC). This morphologically distinct and rarely observed form of invasive ductal carcinoma comprises small and moruliform-like clusters of cancer cells surrounded by clear stromal spaces (cystic areas), and it exhibits lymphotropism and aggressive behavior [1, 2]. Histopathologically, IMPC is characterized by multiple microcystic formations filled with nests of epithelial cells lacking fibrovascular cores [3, 4]. Previous studies evaluating canine IMPC showed several similarities to the corresponding human disease. For example, nests of epithelial cells with a moruliform pattern, a high histological grade, a high incidence of lymph node metastasis and low overall survival rates were observed [5–8]. These and other observations indicated that the canine mammary gland might be an adequate model for pathological comparison with human breast cancer [9].

Emerging evidence shows differences in the IMPC tumor microenvironment that contribute to the mechanisms generating suppressive or tolerant environments that allow tumor regression or progression [10, 11]. The tumor microenvironment comprises tumor cells and surrounding nontumor cells, blood vessels, extracellular matrix (ECM) and various biologically active molecules derived from tumor and nontumor cells [11]. The heterogeneity, dynamic localization and differentiation of mammary gland cells outlining IMPCs are still poorly understood. Furthermore, the transformation of carcinomatous areas in situ to invasive areas requires further study to develop a treatment for canine mammary gland cancer [7, 12, 13]. Tumor progression and the key components involved in that process have been described [14, 15]. IMPC displays unexpected secretory activity at the stroma-facing surface, suggesting a reversal of cell polarity in this type of tumor [1]. For example, the surface glycoprotein mucin 1 (MUC1), which is typically expressed at the apical surface of normal epithelial cells, is reported to play an important role in the detachment of cells from the stroma and facilitates the tumoral spread of cells [2].

In normal breast tissue, a basement membrane separates the ductal epithelium and the underlying myoepithelial cells from the surrounding connective tissue that contains capillaries, fibrillar ECM and fibroblasts [16, 17]. In invasive ductal carcinomas, the basement membrane is ruptured, and the tumor cells often form irregular duct-like areas without a defined basement membrane [18, 19]. The stroma surrounding the tumor cells contains inflammatory infiltrates, newly formed capillaries and myofibroblasts [18].

Stromal cells have been shown to substantially affect normal and tumor tissues and may play a key role in regulating breast epithelial cell function [20]. Resting and activated fibroblasts are associated with cancer cells at all stages of cancer progression. The structural and functional contributions of these cells to cancer progression are beginning to emerge with the definition of cancer-associated fibroblasts (CAFs) [15, 19]. Many molecules are used as CAF markers, for example, alpha-smooth muscle actin (αSMA), an important marker of differentiated myofibroblasts [11, 14, 21]; S100A4, also named fibroblast specific protein-1 (FSP-1), a calcium-binding protein that has been recognized to play a key role in tumor progression and metastasis [22]; and Vimentin, an intermediate filament-associated protein [18, 23, 24].

This study aimed to investigate the neoplastic and stromal cells lining the IMPC cystic space. Immunohistochemical and immunofluorescence (IF) staining were used to assess the presence of cells expressing αSMA, Vimentin, and S100A4. Furthermore, the reversal of epithelial cell polarity was examined in IMPC by investigating the apical glycoprotein MUC1. Finally, transmission electron microscopy (TEM) was utilized to investigate the cells lining the cystic space in canine IMPC. The obtained results may improve our understanding of the canine IMPC microenvironment.

## Results

### Anatomopathological description

**Fig. 1:**
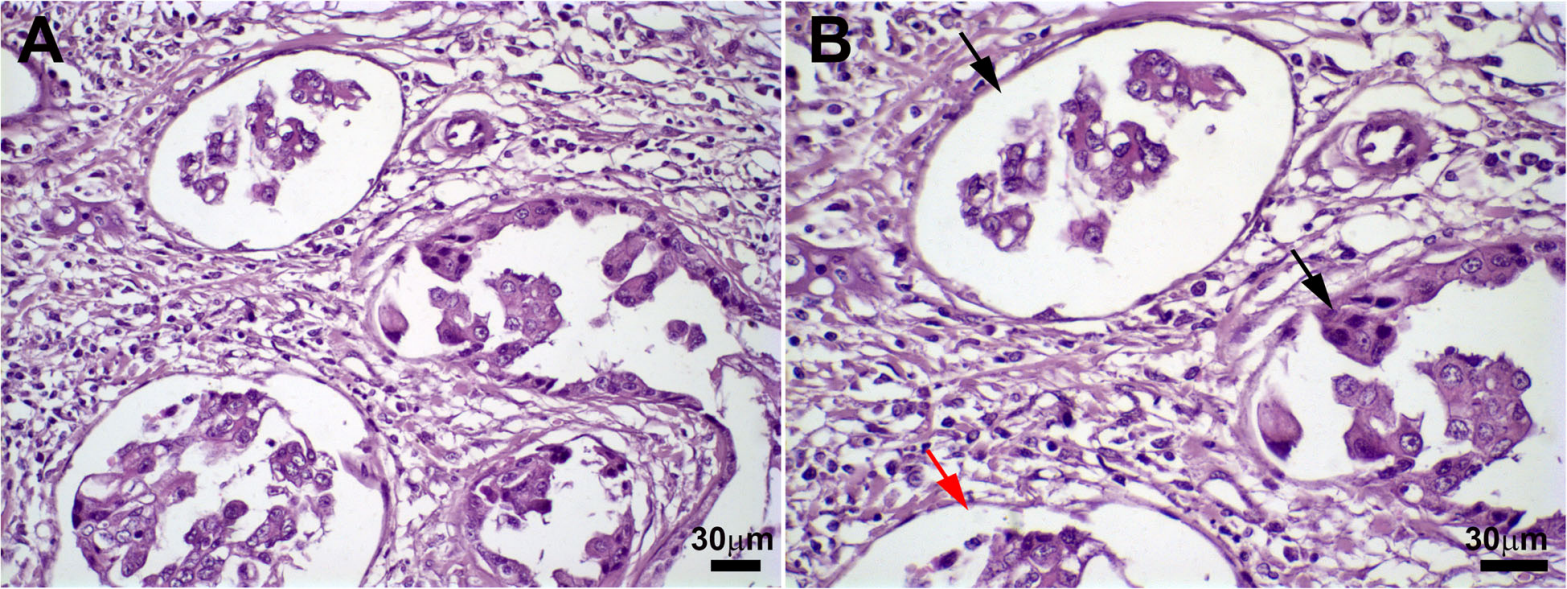
Histopathological appearance of IMPC of the canine mammary gland. Invasive and in situ micropapillary areas characterized by neoplastic epithelial cells within cystic spaces (**a**). An in situ (black arrow) and invasive (red arrows) areas may be observed in detail (**b**) (scale bars = 30 μm). Harris’s hematoxylin staining is shown

IMPC cases were selected from the Laboratory of Comparative Pathology archives (Fig. 1). The age of the animals ranged from 4 to 13 years (mean 10.5 ± 3 years) at the time of surgery. Tumors smaller than 6 cm were predominant (4/8, 50%), and regional metastasis was observed in 62.5% of the dogs (5/8). Regarding the IMPC histopathological analysis, 7 cases were histological grade II, and 1 case was histological grade III. Concerning overall survival, 7 dogs died because of mammary neoplasia, and one dog died due to hemorrhagic diathesis. The overall survival time ranged from 8 to 404 days (median 157 days). Overall survival was defined as the period from surgery to death due to the tumor. The clinicopathological findings and survival data of the dogs are presented in Table 1. One normal canine mammary gland sample was used as a control.

**Table 1:** Clinicopathological and survival findings of the IMPCs of the canine mammary gland

Cases	Age (years)	Breed	Tumor Size (cm)	Lymph node Metastasis	Histological Grade^**a**^	Overall survival (days)^**b**^
**Case 1**	14	Poodle	5	Yes	II	71
**Case 2**	8	Daschound	4	Yes	II	150
**Case 3**	12	Crossbreed	7	Yes	II	30
**Case 4**	8	Akita	4	NA	II	8^§^
**Case 5**	12	English Cocker Spaniel	8	NA	II	150
**Case 6**	13	Poodle	6	Yes	III	404
**Case 7**	11	Crossbreed	11	Yes	II	360
**Case 8**	10	Bichon Frise	2	NA	II	90

*NA* Not Available.

^a^Histological grading based on that of Elston and Ellis (1991, 1998).

^**b**^The overall survival time was defined as the period (in days) between surgery and death due to the tumor. Seven canines evaluated, died due to the disease; nevertheless, in the case 4 (^§^) the dog died because of a hemorrhagic diathesis.

### Immunohistochemical analysis

**Fig. 2:**
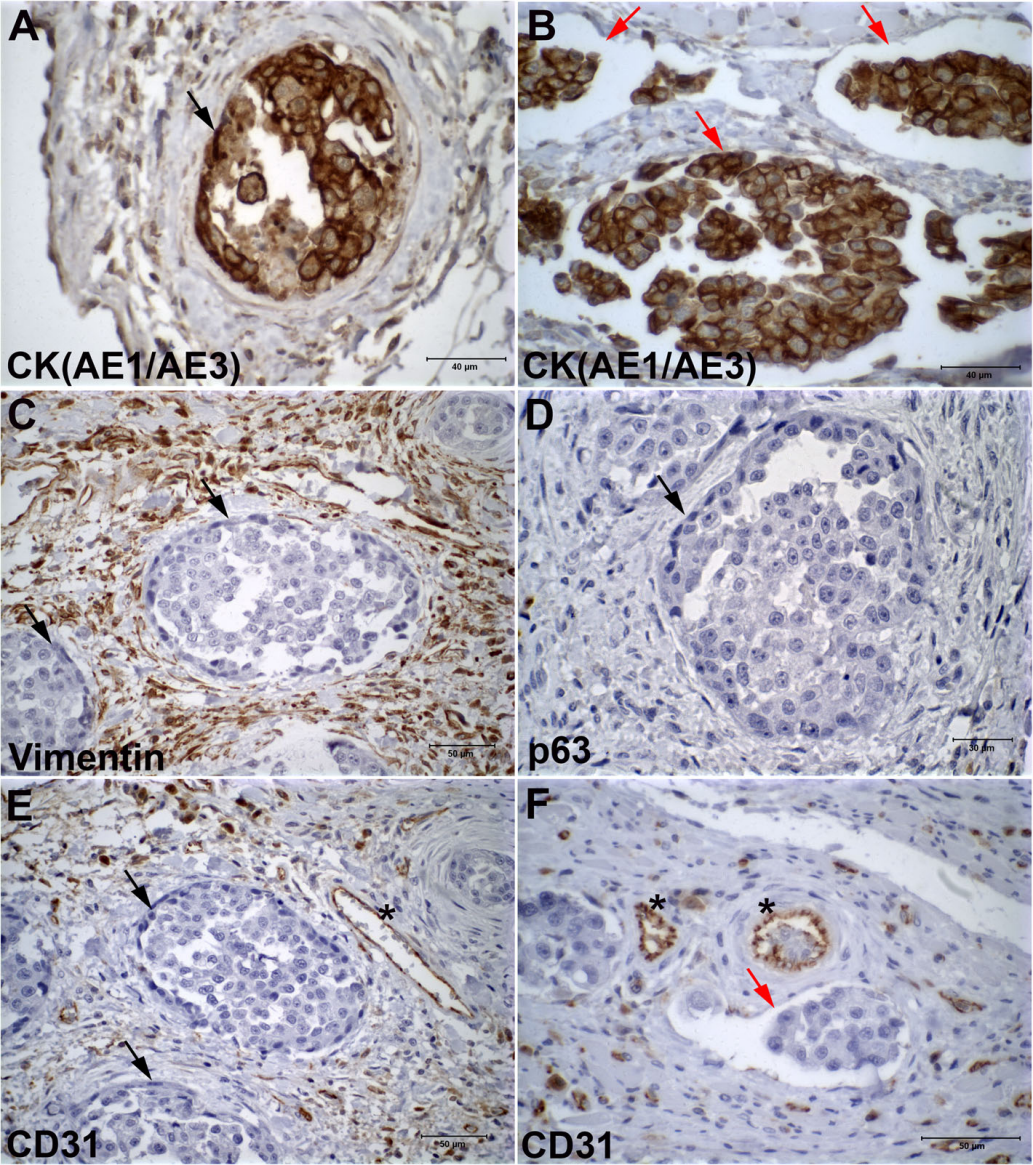
Photomicrographs illustrating Cytokeratin (AE1/AE3), Vimentin, p63, and CD31 immunostaining in IMPC of the canine mammary gland. Epithelial neoplastic cells exhibiting positive staining for Cytokeratin (AE1/AE3) staining in in situ (black arrow) and invasive regions (red arrows) of IMPC (**a-b**) (scale bar = 40 μm). Vimentin-positive labeling confirms the presence of a mesenchymal component in IMPC (**c**) (scale bar = 50 μm). Negative p63 staining in IMPC (**d**) (scale bar = 30 μm). Positive staining for CD31 is detected only in the plasma membrane of endothelial cells (see asterisks) (**e-f**) (scale bar = 50 μm). In situ regions are shown with black arrows and invasive regions with red arrows. (Novolink™ Polymer Detection System, counterstained with Harris’s hematoxylin)

Immunohistochemical analyses were performed in all primary IMPCs, and images were acquired using a light microscope. All IMPC samples contained numerous irregular cystic formations with nests of epithelial cells in a moruliform pattern (Fig. 1). The lumen exhibited cell clusters in the in situ areas, indicating a transition to invasive areas, and the neoplastic cells were pleomorphic with a typical polygonal morphology. We observed Cytokeratin (CK) (clone AE1/AE3) staining, indicating the epithelial cells in IMPCs (Fig. 2a and b). We used Vimentin staining to investigate the cellular localization of the stromal cells outlining the IMPCs and observed cytoplasmic staining indicating mesenchymal cells around the IMPC areas (Fig. 2c). P63 staining for myoepithelial cells was observed to be negative in IMPCs (Fig. 2d); CD31 staining was positive in the tumor-associated vessels around the tumor but was not observed in IMPC cystic formations (Fig. 2e and f). Normal canine mammary gland tissue was used as a positive control. In this sample, the ductal epithelial cells were positive for CK, myoepithelial cells were positive for p63, blood vessel endothelial cells were positive for CD31, and the mammary gland stromal areas were positive for Vimentin (see Supplementary Fig. 1). Collectively, these results document the presence of epithelial cells (CK^+^) and mesenchymal cells (vimentin^+^) and the absence of myoepithelial (p63^−^) and endothelial (CD31^−^) cells in IMPC. No differences in immunostaining were observed between the in situ and invasive areas.

### Phenotypic characterization of stromal and neoplastic cells using superresolution microscopy

A Zeiss LSM 880 with an Airyscan detector was used to overcome the limited image resolution and improve the signal-to-noise ratio compared with those of conventional light microscopes and confocal systems. This system utilizes deconvolution by properly weighting the signals from a combination of 32 detectors to improve the three-dimensional (3D) resolution and has been applied in biomedical research and clinical diagnosis [25]. Observation of immunohistochemical staining does not allow precise colocalization of signals, and multiple colors cannot be visualized simultaneously in 3D [26].

**Fig. 3:**
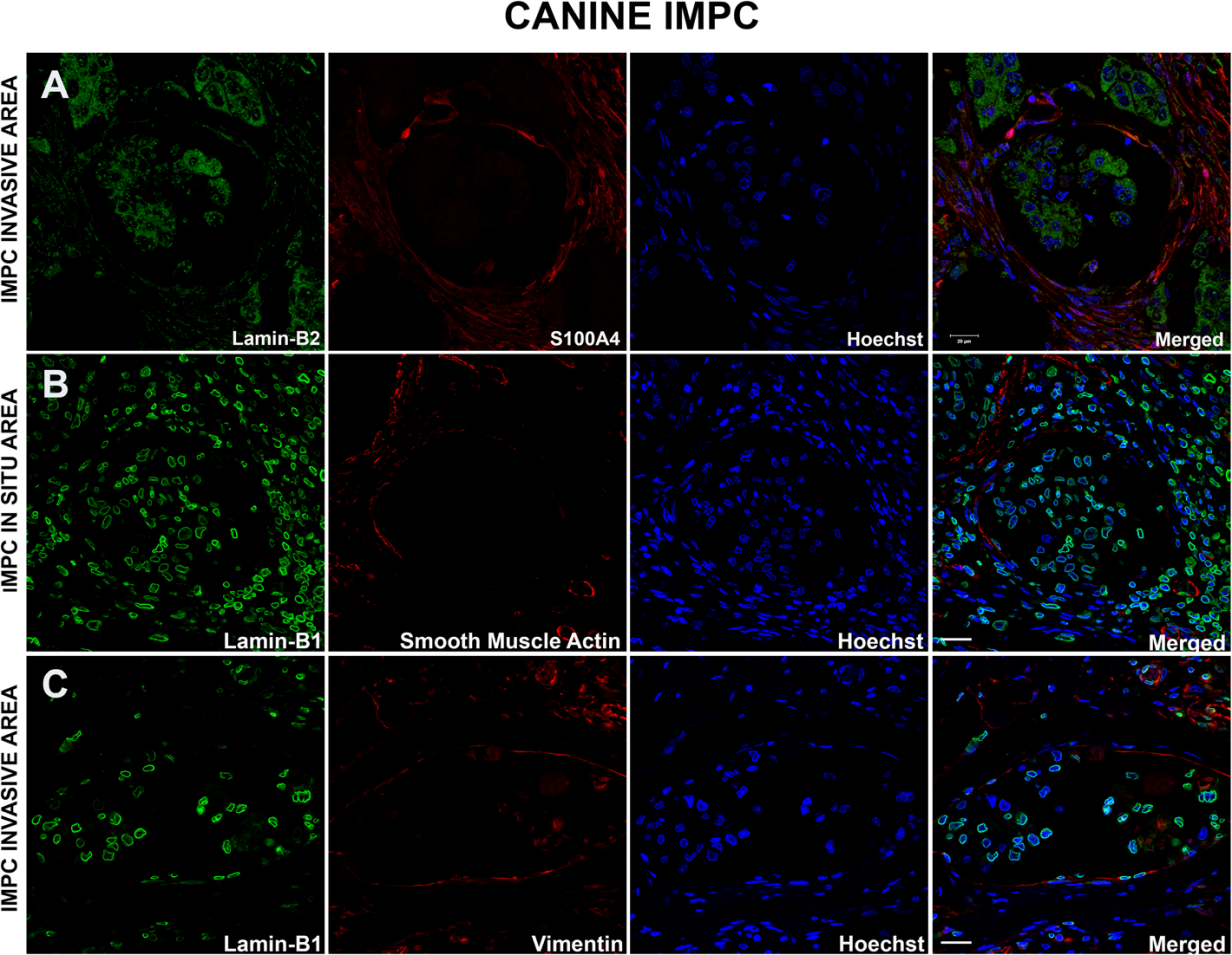
S100A4, αSMA, and vimentin expression in invasive areas in an IMPC of the canine mammary gland. Superresolution images of the nuclear envelope markers Lamin B1/B2 (green), S100A4, αSMA, and Vimentin (red); nuclei were stained with Hoechst (blue) in invasive (**a-c**) and in situ areas (**b**). Note that intermediate microfilament staining surrounds the cystic space of micropapillary tumoral areas. The merged images show the overlapping staining. Representative images from *n* = 4 discrete primary tumor cases are shown, and ten images were acquired from invasive areas in each sample. Scale bars = 20 μm

**Fig. 4:**
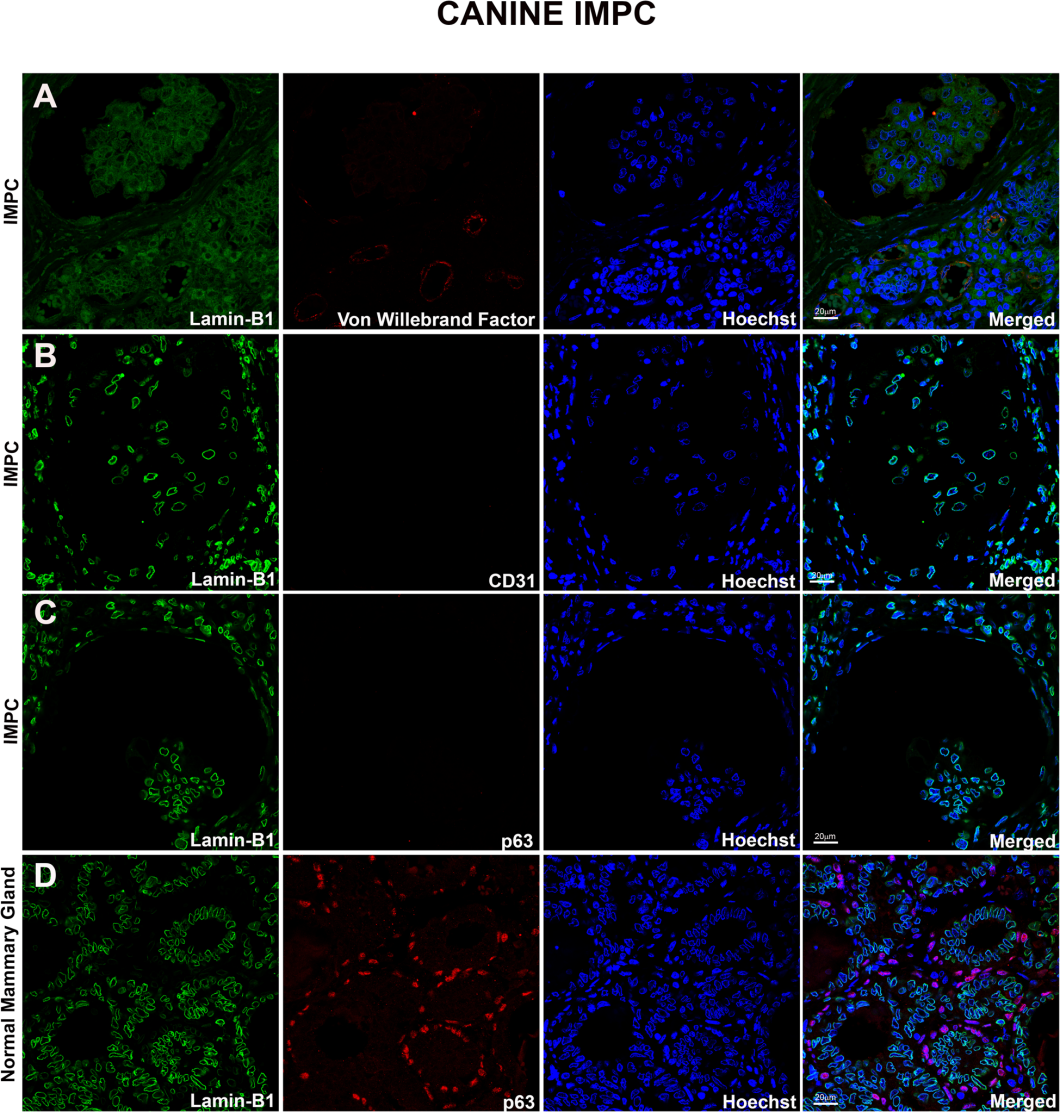
Negative staining for von Willebrand factor, CD31, and p63 in the cystic spaces of IMPC areas in the canine mammary gland. Superresolution images of the nuclear envelope markers Lamin B1/B2 (green), von Willebrand factor (**a**), CD31 (**b**), and p63 (**c**) (red); nuclei were stained with Hoechst (blue). Note that the staining is negative for endothelial and lymphatic cell markers (CD31 or von Willebrand factor) in micropapillary areas. Staining for the myoepithelial cell marker p63 is negative in IMPC and positive in the control sample of a normal mammary gland (**d**). The merged images show the overlapping signals. Representative images from *n =* 4 discrete primary tumor cases are shown, and ten images were acquired from in situ and invasive areas in each case. Scale bars = 20 μm

**Fig. 5:**
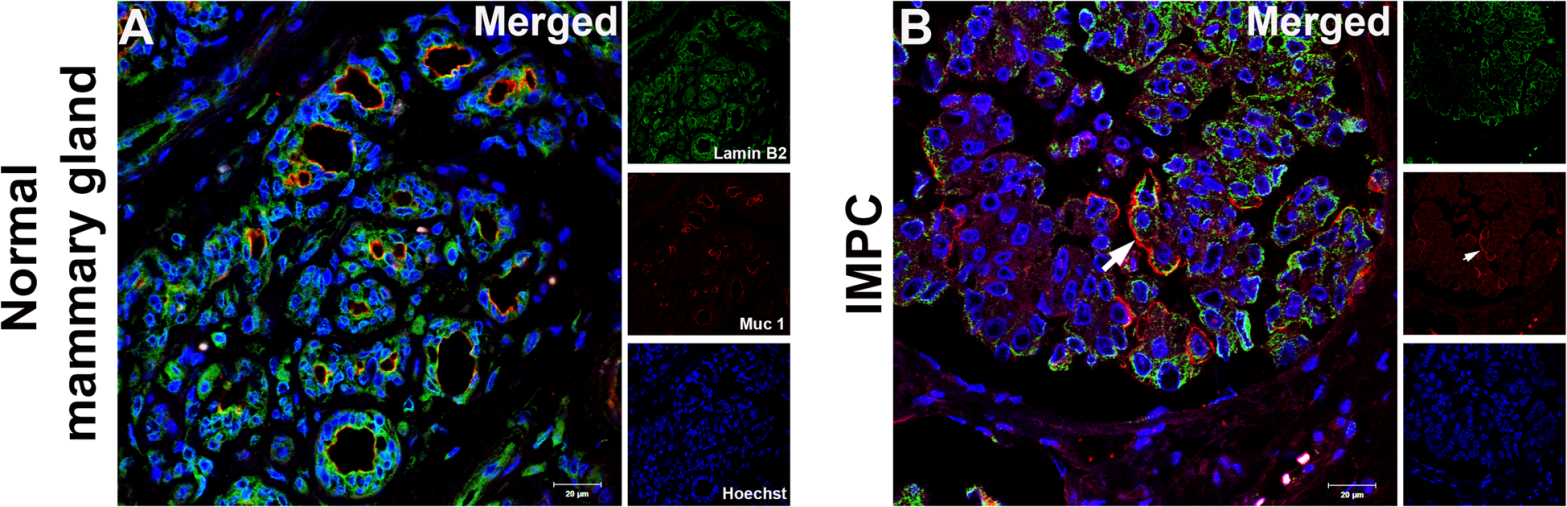
MUC1 expression in IMPC of the canine mammary gland. Superresolution merged images show the overlapping signals for the nuclear envelope markers Lamin B1/B2 (green) and MUC1 (red); nuclei were stained with Hoechst (blue). **a** The MUC1 positive control shows apical staining in the healthy mammary gland. **b** Positive staining is observed on the stroma-facing surface of the canine IMPC samples (white arrows). Representative images from 4 cases of IMPC of the canine mammary gland are shown, and ten images were acquired from invasive areas. Scale bars = 20 μm

We performed double labeling of S100A4, αSMA or Vimentin with Lamin B1/B2 in all micropapillary samples from canine mammary glands to observe the expression of these markers in the stromal and neoplastic cells outlining the IMPC cystic space. Lamin B1/B2 was used as a marker of the inner nuclear membrane (INM). The IMPC samples exhibited numerous irregular cystic formations containing nests of epithelial cells in a moruliform pattern. In all eight IMPC samples evaluated, cells were positive for S100A4, SMA, and Vimentin in invasive and in situ areas (Fig. 3a, b and c and Supplementary Fig. 2a, b and c). We observed the same labeling pattern on stromal cells in the human IMPC sample used as the positive control (see Supplementary Fig. 4a, b, and c). Staining for von Willebrand factor and CD31 was observed in the plasma membrane of lymphatic and endothelial cells, but these markers were not observed in the IMPC cystic formations. This control confirmed the true nature of the IMPC cases in this study (Fig. 4a and b, Supplementary Fig. 3a and b, and Supplementary Table 1). Similarly, staining for von Willebrand factor and CD31 was not detected in the cystic area of the human IMPC, confirming our previous observation (see Supplementary Fig. 5a and b). Serial superresolution images showed the localization of the stromal cells surrounding the epithelial nests (Figs. 3 and 4). Furthermore, staining for the myoepithelial cell marker p63 was negative in IMPC samples and positive in normal mammary glands used as positive controls (see Fig. 4c and d and Supplementary Fig. 3c and d). Similar results were obtained for the human IMPC (see Supplementary Fig. 5c). We performed MUC1 staining to investigate the reversed polarity of IMPC cells. Positive and diffuse MUC1 staining was detected on the stroma-facing (basal) surface of the neoplastic cell clusters in all IMPCs of the canine mammary gland (Fig. 5a and b and Supplementary Fig. 2d). Diffuse MUC1 staining was observed in the human IMPC sample, and this labeling confirmed the previous results (see Supplementary Fig. 4d). Collectively, these results are the first to characterize the stromal cells surrounding the IMPC cystic space in the canine mammary gland using superresolution microscopy and IF staining.

### Ultrastructure of normal and IMPC myoepithelial cells

**Fig. 6:**
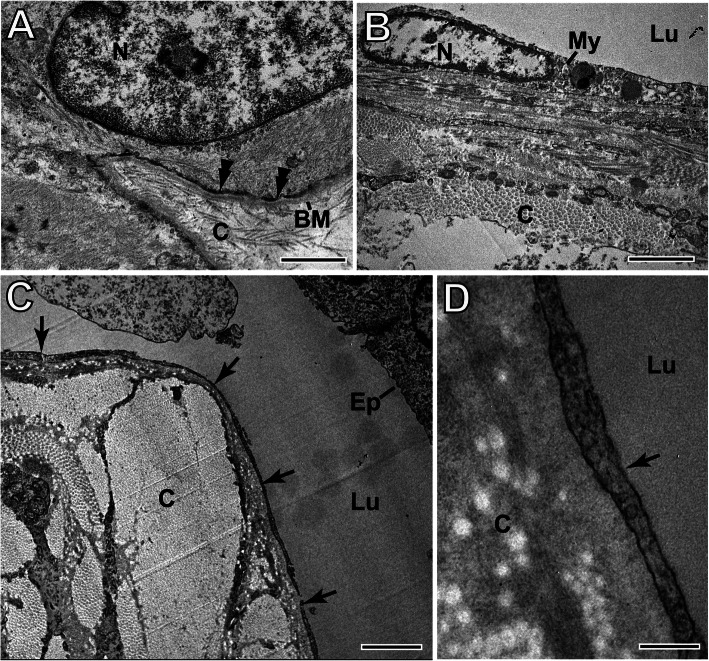
Ultrastructures of normal and canine IMPC myoepithelial cells. **a** Normal myoepithelial cells show ovoid nuclear (N) shapes and hemidesmosomes (double arrowheads) along the evident basement membrane (BM). In IMPC (**b**), myoepithelial-like cells (My) show a smaller and flatter nucleus and thinner cytoplasm. In (**c**) and (**d**), thin cytoplasmic projections of My cells (arrows) coating the large lumen (Lu) of the cystic space located close to dense and ordered bundles of collagen fibrils are observed (**c**). Ep, neoplastic epithelial cell. Scale bars: a and b = 1 μm; c = 2 μm; d = 200 nm

The normal myoepithelial cells showed a thin elongated cytoplasm with tapering bipolar processes. Their nuclei had marked invaginations or notches in the nuclear membrane and predominant heterochromatin. The cytoplasm was filled with few organelles and a large amount of myofilaments. The myoepithelial cells were connected to the stroma by hemidesmosomes along the evident basement membrane (Fig. 6a). These myoepithelial-like cells exhibited smaller and flatter oval nuclei without notches and with predominant euchromatin. The cytoplasm was observed as very thin, and long cytoplasmic extensions with a reduced amount of myofilaments and the absence of microvilli, intercellular junctions and basement membrane (Fig. 6b and c). Neoplastic myoepithelial cells had long and thin cytoplasmic extensions outlining the large luminal area of the invasive cystic space of IMPCs (Fig. 6d and e). In IMPCs, the morphology of myoepithelial cells was modified. Similar myoepithelial-like cells were observed in in situ IMPC regions (data not shown).

## Discussion

IMPC of the breast is a morphologically distinct form of invasive ductal carcinoma that occurs in women [2]. The constituent tumor cells are typically arranged in small clusters with a central lumen, and micropapillae lacking fibrovascular cores extend into clear spaces [2, 6, 27]. This type of cancer in women is extremely aggressive, and based on previous studies from our group, it appears to exhibit a similar behavior in dogs [1, 5, 6, 8]. In the present study, we performed morphological analysis using immunohistochemical and IF staining and ultrastructural analysis using TEM to characterize the neoplastic cells outlining the cystic space of IMPC in the canine mammary gland.

The tumor microenvironment contains several cell types, including endothelial cells, fibroblasts, myofibroblasts, and other cells. The stroma around invasive breast tumors is described to differ from normal breast tissue, with alterations in stromal protein synthesis [20]. Compared to normal tissues, CAFs express increased levels of proteins such as αSMA and S100A4 [11]. Fibroblasts are embedded within the fibrillar ECM of the connective tissue and constitutively express Vimentin and fibroblast-specific protein 1 [18, 28]. Several differences in the stroma of invasive breast cancer are attributed to activated fibroblasts, also termed myofibroblasts, reflecting their acquisition of αSMA expression [29, 30]. In our study, we observed stroma-associated fibroblasts. We detected αSMA expression in IMPC cystic areas; αSMA expression may be a marker for increased aggressiveness of this type of tumor. Indeed, SMA expression and acquisition have been reported to be a prerequisite for tumor invasiveness in breast cancer [31]. S100A4 has been observed to be overexpressed in advanced-stage thyroid carcinoma and in patients with breast, gastric, pancreatic, lung, and prostate cancers [22, 32–34].

S100A4 expression was positive in both invasive areas of the tumor transition zone (in situ to invasive) in the present study. This protein may play a role in other advanced diseases with lymph node metastasis [35, 36]. We also observed positive staining for the intermediate filament-associated protein vimentin in canine IMPC. Typically, fibroblasts appear as fusiform cells with a prominent actin cytoskeleton and intermediate filaments composed of vimentin [18]. Fibroblasts participate in the repair process by differentiating into activated myofibroblasts, which help maintain the inflammatory response to injury [28, 37, 38]. Myofibroblasts are large cells with a highly active endoplasmic reticulum, and they can produce and secrete cytokines [37]. Other groups have reported roles for fibroblasts in the initiation of cancer invasion, and these cells have been termed CAFs [11, 38]. These cells are reported to be atypical tumor stromal fibroblasts and are related to tumor progression and recurrence [39, 40]. CAFs promote ECM remodeling, proliferation, invasion, and inflammation and are considered to play key roles in the tumor microenvironment [41, 42]. During epithelial-to-mesenchymal transition, epithelial cells are converted into fibroblast-like cells in various tissues, and this process requires further investigation in canine tumors. Fibroblasts might also play a role in metastasis, and therapies targeting CAFs are being considered for cancer control [18].

In our study, all canine IMPC cases exhibited numerous irregular stromal cystic formations containing nests of epithelial cells in a moruliform pattern. Positive staining for CD31 and von Willebrand factor was observed in the plasma membrane of only endothelial and lymphatic cells of tumor-associated vessels in the peritumoral region but was not observed in the IMPC cystic formations. The empty spaces outlining the micropapillae of the carcinoma cell nests were not surrounded by endothelial and myoepithelial cells, thus confirming the infiltrative micropapillary nature of the tumors [6, 43].

According to Tavassoli (1999), the most important feature for distinguishing a papilloma from a papillary carcinoma is the presence of a relatively uniform myoepithelial cell layer in the proliferating intraluminal component of the lesion, and the absence of the basal myoepithelial layer in the papillary processes almost always indicates a carcinoma [44]. Several newer markers, including p63, have been used to successfully identify myoepithelial cells. Histological or immunolocalization-based identification of a myoepithelial cell layer has become a key feature used to distinguish benign from malignant and in situ from invasive lesions of the breast [45–47]. P63 shows no cross-reactivity with myofibroblasts or vascular smooth muscle cells [45]. Other studies have identified p63 as a sensitive and highly specific marker of myoepithelial cells in canine mammary tissues [48]. Differences in indicators of malignancy between luminal epithelial cell types and myoepithelial cells have been reported [49]. In our study, we observed negative staining for p63 in all IMPC cases analyzed (see Fig. 4), although TEM observation revealed myoepithelial-like cells covering the cystic space. By IHC, Gamba et al. (2013) documented positive nuclear p63 staining in 20% of IMPCs of the canine mammary gland [6]. In our study, careful background adjustments in the IF staining images based on the negative controls decreased the chances of detecting false positive signals in the immunohistochemical staining samples.

In histopathological evaluations via light microscopy, IMPC cystic spaces do not appear to have a visible cell coating, as previously observed [44]. However, when this region was evaluated by TEM, it was observed, for the first time in canines, the presence of cells morphologically similar to myoepithelial cells. Still, under certain aspects, they differ from the normal mammary gland [17]. In addition to flattened nuclei without membrane notches and long and thin cytoplasmic extensions in the nucleoplasm, these cells exhibited predominant euchromatin. These ultrastructural characteristics, the absence of notches in the nuclear membrane and the abundant euchromatin (higher DNA synthesis) indicate malignancy [17]. These TEM observations and the absence of p63 suggested that these cells were neoplastic myoepithelial-like cells. In humans, although p63 was expressed in myoepithelial cells in benign lesions, the loss of expression is indicative of malignant lesions associated with invasive progression [50, 51].

IMPCs also show unexpected secretory activity from the stroma-facing surface of tumor cells, suggesting a reversal of cell polarity. Other groups have investigated the distribution of MUC1, which is expressed on the apical surface of several types of epithelial tissues [2, 52]. Nassar et al. (2004) showed the distribution of MUC1 in human IMPCs from different organs. This study characterized a reversal of cellular polarization and expression of MUC1 in the stroma-facing surface of the cells [2]. Other markers, such as E-selectin and its ligand sialyl Lewis X (SLX), may play a role in lymph node metastasis in IMPC, and the expression pattern of SLX suggested the reversal of cell polarity in IMPC [13, 53]. We report a new observation of MUC1 expression in the stroma-facing surface of canine tumor cells. Our IF staining and superresolution microscopy results confirmed the reversal of cell polarity, and our findings were similar to those observed in human IMPC tissue. MUC1 expression on the stroma-facing surface of the cells might be a key factor contributing to the distinct morphology of this tumor type by causing the detachment of neoplastic cells from the stroma [2].

## Conclusions

The present study provides a morphological, immunohistochemical, and ultrastructural characterization of the neoplastic cells outlining the cystic space of IMPC. The cells expressing Vimentin, S100A4, and αSMA are likely to be resting or activated fibroblasts that may play an important role in this type of neoplasia. For the first time, the cystic space of canine IMPC was shown to be delimited by thin extensions of myoepithelial-like cells that have lost p63 expression. These findings may help to improve IMPC treatment and diagnosis.

## Methods

### Case selection, histopathological analysis, and overall survival analysis

Samples from eight cases of IMPC and one control normal canine mammary gland were selected at the Laboratory of Comparative Pathology of the Institute of Biological Sciences, Universidade Federal de Minas Gerais (UFMG), after approval by the Animal Experimentation Ethics Committee (CEUA protocol number: 362/2016). The human IMPC sample used as a positive control was donated by the Laboratory of Clinical Analysis “Hermes Pardini”, Belo Horizonte, Minas Gerais, and was approved by the Research Ethics Committee (CAAE-0002.0.204.203–11).

For histopathological analysis (grading, histotype and margins), primary tumor specimens were fixed with 10% neutral buffered formalin, embedded in paraffin (to generate formalin-fixed, paraffin-embedded (FFPE) samples), sliced into 4 μm thick histological sections, and stained with hematoxylin and eosin (Sigma-Aldrich, Carlsbad, CA, USA). All cases were reviewed and reclassified independently by two pathologists (GDC and TS). In brief, carcinomas with cystic formations containing nests of epithelial cells with a moruliform appearance (an infiltrating micropapillary pattern) were diagnosed as IMPC, regardless of whether they were associated with in situ micropapillary areas [54] (Fig. 1). The invasive areas of canine IMPCs were graded according to the Nottingham grading system [55, 56]. The overall survival time was defined as the period (in days) between surgery and death due to the tumor. The follow-up period was 400 days. Seven of the dogs evaluated died due to the disease; however, in one case, the dog died because of hemorrhagic diathesis. The survival rate was calculated using the Kaplan–Meier method [6, 9, 57].

### Immunohistochemistry

IHC was performed as previously described with minor modifications [9, 27]. Sections (4 μm) of primary tumors were mounted on silanized slides, and a peroxidase-based detection system, Novolink™ Polymer (Leica Biosystems Newcastle Ltd., Newcastle, UK), was applied. Slides were dewaxed in xylene, and endogenous peroxidase activity was quenched with 3% H_2_O_2_ in methanol. The reagents were applied manually, and immunoreactivity was visualized by incubating the slides with 3,3′-diaminobenzidine (Lab Vision DAB substrate system; Lab Vision, Fremont, California, USA) for 5 min. The antibodies used in this study are described in detail in Table 2. Negative controls were established using normal serum (Lab Vision Ultra V Block) instead of the primary antibody. Normal canine mammary gland tissue was used as a positive control for all immunohistochemical staining procedures (see Supplementary Fig. 1).

**Table 2:** Details of the antibodies used for immunohistochemistry and immunofluorescence in canine mammary tumors

ANTIBODY	SOURCE	CLONE	DILUTION	MANUFACTURER	ANTIGEN RETRIEVAL	INCUBATION
CD31	mouse	JC70A	1:100	DAKO	Pressurized Heating (125°)	Overnight (4°)
Cytokeratin	mouse	AE1/AE3	1:100	DAKO	Pressurized Heating (125°)	Overnight (4°)
Factor VIII- associated factor	rabbit	polyclonal	1:800	DAKO	Pressurized Heating (125°)	Overnight (4°)
Lamin B1	rabbit	polyclonal	1:100	ABCAM	Pressurized Heating (125°)	Overnight (4°)
Lamin B2	mouse	monoclonal	1:100	ABCAM	Pressurized Heating (125°)	Overnight (4°)
MUC1	rabbit	EP1024Y	1:200	ABCAM	Pressurized Heating (125°)	Overnight (4°)
αSMA	mouse	1A4	1:100	DAKO	Pressurized Heating (125°)	Overnight (4°)
S100A4	rabbit	polyclonal	1:100	Thermo Scientific	Pressurized Heating (125°)	Overnight (4°)
p63	mouse	4A4	1:100	Neomarkers	Pressurized Heating (125°)	Overnight (4°)
Vimentin	mouse	3B4	1:100	DAKO	Pressurized Heating (125°)	Overnight (4°)

### IF staining for phenotypic markers and imaging via superresolution microscopy

IF staining was performed as previously described by Rodrigues M.A. et al. (2016) [12, 58–60]. In brief, FFPE tissue sections were dewaxed, rehydrated, and unmasked in Trilogy solution (Cell Marque, Koclin, CA, USA) with pressurized heating (125 °C) for 20 min according to the manufacturer’s instructions. Next, samples were rinsed with phosphate-buffered saline (PBS, 137 mM NaCl, 2.7 mM KCl and 10 mM phosphate buffer solution (pH 7.4)) (Sigma-Aldrich), incubated with PBS containing 0.2% Triton X-100 (Sigma-Aldrich) for another 20 min, and blocked with PBS containing 1% bovine serum albumin (BSA, Sigma-Aldrich) for 30 min. Next, sections were incubated with primary antibodies against the nuclear envelope markers Lamin B1/B2, αSMA, Vimentin, CD31, von Willebrand factor, p63, S100A4 and MUC1. Detailed information about each antibody is presented in Table 2. The immunoreactivity of these antibodies has already been validated in canine species, as described in previous studies and by the manufacturer [6, 24, 61–67]. One sample of human IMPC was used as a positive control for all labeling performed in the present study (see Supplementary Figs. 4 and 5). Then, sections were rinsed three times with PBS for 5 min each. Subsequently, sections were incubated with an Alexa Fluor® 488-conjugated goat anti-rabbit IgG antibody (1:1000, Life Technologies, Carlsbad, CA, USA), an Alexa Fluor® 555-conjugated goat anti-mouse IgG antibody (1:1000, Life Technologies) and Hoechst 33258 (1 μg/mL, Life Technologies) for 1 h at room temperature. Next, the samples were washed 3x with PBS for 10 min each and were then mounted using Prolong Gold Antifade Reagent (Life Technologies). A negative control was included in all reactions by omitting the primary antibodies (see Supplementary Fig. 3e). Images were acquired with a Zeiss LSM 880 connected to an Airyscan detector (Carl Zeiss, Jena, Germany) using an 40× 1.3 NA oil objective. In this study, we used the Airyscan system to increase the signal-to-noise ratio and resolution. This system is a 32-channel GaAsP-PMT area detector that is used for superresolution microscopy to resolve structures beyond the diffraction-limited resolution of conventional light microscopes [68]. Samples were excited at 405 nm and observed at 420–480 nm to detect Hoechst, excited at 488 nm and observed at 500–525 nm to detect Alexa Fluor 488, and excited at 543 nm and observed using a longpass (LP) 570 nm filter to detect the Alexa Fluor 555 signal. Zeiss Efficient Navigation (ZEN) software was used to display orthogonal projections (XY, XZ, and YZ). The fluorescence microscopy results were evaluated in invasive areas of IMPCs, and five to ten images were acquired from each sample. Seven markers were analyzed (*n* = 300 images).

### Tissue processing for ultrastructural evaluation

For TEM, one normal and one tumor biopsy sample (five fragments of each) fixed with 10% neutral buffered formalin were cut into pieces of approximately 2 mm (length x width) and subsequently postfixed with 5% glutaraldehyde (biological grade; Electron Microscopy Sciences, Hatfield, PA, USA) in 0.05 M phosphate buffer (pH 7.3) for 24 h. Then, the fragments were postfixed with reduced osmium (osmium tetroxide 1% and potassium ferrocyanide in distilled water) for 90 min and dehydrated in ethanol and acetone before embedding in epoxy Araldite resin (Electron Microscopy Sciences, Hatfield, PA, USA). After preparation of ultrathin sections at a thickness of 60 nm, images were acquired using a Tecnai G-12 FEI–120 KV microscope. The images were adjusted for resolution, sharpness, and contrast using Adobe Photoshop software (Adobe System, Inc., Mountain View, CA, USA).

## Supplementary Information

**Additional file 1: Supplementary Fig. 1** Photomicrographs illustrating p63, CD31, Vimentin and Cytokeratin (AE1/AE3) immunostaining in normal canine mammary glands. **(a)** Positive staining for normal myoepithelial cell markers is observed in cells lining the mammary gland ducts. **(b)** Positive staining for CD31 on the plasma membrane of endothelial cells. **(c)** Positive staining for Vimentin in stromal cells. **(d)** Representative image of IHC staining in the negative control (NC) sample. **(e)** Positive staining for CK (AE1/AE3) in ductal epithelial cells. Scale bars = 20 μm and 50 μm. (Novolink™ Polymer Detection System, counterstained with Harris’s hematoxylin).

**Additional file 2: Supplementary Fig. 2** Representative images of S100A4, αSMA, Vimentin and MUC1 expression in invasive IMPC areas of the canine mammary gland. **(a-c)** Superresolution images of the nuclear envelope markers Lamin B1/B2 (green), S100A4, αSMA, and Vimentin (red); nuclei were stained with Hoechst (blue) in invasive areas. **(d)** Stroma-facing positive staining for MUC1 (red) in canine IMPCs. The merged images show the overlapping signals. Representative images from *n* = 4 discrete primary tumor cases are shown, and 5 images were acquired from invasive areas in each sample. Scale bars = 20 μm.

**Additional file 3: Supplementary Fig. 3** Negative staining for von Willebrand factor, CD31, and p63 in cells outlining the cystic spaces of IMPC areas in the canine mammary gland. **(a)** Superresolution images of the nuclear envelope markers Lamin B1/B2 (green), von Willebrand factor, CD31 **(b),** and p63 **(c)** (red); nuclei were stained with Hoechst (blue). Note that staining is negative for endothelial and lymphatic cell markers (CD31 and von Willebrand factor) in micropapillary areas. Staining for the myoepithelial cell marker p63 is positive in the normal mammary gland control sample **(d)**. The merged images show the overlapping signals. Representative image of IF staining in the negative control sample **(e)**. Representative images from *n =* 4 discrete primary tumor cases are shown, and 5 images were acquired from invasive areas in each sample. Scale bars = 20 μm.

**Additional file 4: Supplementary Fig. 4** Representative images of S100A4, αSMA, Vimentin and MUC1 expression in invasive areas of human IMPCs. **(a-c)** Superresolution images of the nuclear envelope markers Lamin B1/B2 (green), S100A4, αSMA, and Vimentin (red); nuclei were stained with Hoechst (blue) in invasive areas. **(d)** Stroma-facing positive staining for MUC1 (red) is observed in human IMPC. The merged images show the overlapping signals. Representative images from one primary tumor case are shown, and 5 images of staining with each antibody were acquired from invasive areas. Scale bars = 20 μm.

**Additional file 5: Supplementary Fig. 5** Negative staining for von Willebrand factor, CD31, and p63 in cells outlining the cystic spaces of invasive micropapillary areas in the human breast. **(a)** Superresolution images of the nuclear envelope markers Lamin B1/B2 (green), von Willebrand factor, CD31 **(b),** and p63 **(c)** (red channel); nuclei were stained with Hoechst (blue). Note that the staining is negative for endothelial and lymphatic cell markers (CD31 or von Willebrand factor) in micropapillary areas. **(d)** Representative images of IF staining in the negative control sample. The merged images show the overlapping signals. Representative images from one primary tumor case are shown, and 5 images of staining with each antibody were acquired from invasive areas. Scale bars = 20 μm.

**Additional file 6: Table 1**

## Data Availability

The datasets used during the current study are available from the corresponding author upon reasonable request.

## Abbreviations

IMPC: Invasive micropapillary carcinoma
IHC: Immunohistochemistry
IF: Immunofluorescence
TEM: Transmission electron microscopy
CAFs: Cancer-associated fibroblasts
FFPE: Formalin-fixed, paraffin-embedded
INM: Inner nuclear membrane
αSMA: α-smooth muscle actin
S100A4: Fibroblast specific protein-1 (FSP-1)
MUC1: Mucin 1
CD31: Cluster of differentiation 31
CK: Cytokeratin
p63: Transformation-related protein 63
LP: Longpass
ZEN: Zeiss Efficient Navigation
BSA: Bovine serum albumin

## References

[1] Luna-Moré S, Gonzalez B, Acedo C, Rodrigo I, Luna C (1994). Invasive micropapillary carcinoma of the breast. Pathol Res Pract.

[2] Nassar H, Pansare V, Zhang H, Che M, Sakr W, Ali-Fehmi R, Grignon D, Sarkar F, Cheng J, Adsay V (2004). Pathogenesis of invasive micropapillary carcinoma: role of MUC1 glycoprotein. Mod Pathol.

[3] Siriaunkgul S, Tavassoli FA (1993). Invasive micropapillary carcinoma of the breast. Mod Pathol.

[4] De La Cruz C, Moriya T, Endoh M, Watanabe M, Takeyama J, Yang M (2004). Invasive micropapillary carcinoma of the breast: Clinicopathological and immunohistochemical study. Pathol Int.

[5] Cassali GD, Serakides R, Gärtner F, Schmitt FC (2002). Invasive micropapillary carcinoma of the dog mammary gland: a case report. Arq Bras Med Veterinária e Zootec.

[6] Gamba CO, Dias EJ, Ribeiro LGR, Campos LC, Estrela-Lima A, Ferreira E, Cassali GD (2013). Histopathological and immunohistochemical assessment of invasive micropapillary mammary carcinoma in dogs: a retrospective study. Vet J.

[7] Gamba CO, Campos LC, Negreiros-Lima GL, Maciel-Lima K, Sousa LP, Estrela-Lima A, Ferreira E, Cassali GD (2014). ZEB2 and ZEB1 expression in a spontaneous canine model of invasive micropapillary carcinoma of the mammary gland. Res Vet Sci.

[8] Cassali GD, Gärtner F, Vieira da Silva MJ (1999). Schmitt FC Cytological diagnosis of a metastatic canine mammary tumor in pleural effusion. Arq Bras Med Veterinária e Zootec.

[9] de Oliveira GC, Scaratti D, de Andrade VP, Estrela-Lima A, Ferreira E, Cassali GD (2017). Invasive micropapillary carcinoma of the mammary gland in humans and canines: Clinicopathological, immunophenotypical and survival approaches. Res Vet Sci.

[10] Hanahan D, Coussens LM (2012). Accessories to the crime: functions of cells recruited to the tumor microenvironment. Cancer Cell.

[11] Bussard KM, Mutkus L, Stumpf K, Gomez-Manzano C, Marini FC (2016). Tumor-associated stromal cells as key contributors to the tumor microenvironment. Breast Cancer Res.

[12] Gamba CO, Rodrigues MA, Gomes DA, Estrela-Lima A, Ferreira E, Cassali GD (2015). The relationship between E-cadherin and its transcriptional repressors in spontaneously arising canine invasive micropapillary mammary carcinoma. J Comp Pathol.

[13] Yang Y-L, Liu B-B, Zhang X, Fu L (2016). Invasive micropapillary carcinoma of the breast: an update. Arch Pathol Lab Med.

[14] De Wever O, Demetter P, Mareel M, Bracke M (2008). Stromal myofibroblasts are drivers of invasive cancer growth. Int J Cancer.

[15] Park CK, Jung WH, Koo JS (2016). Expression of cancer-associated fibroblast-related proteins differs between invasive lobular carcinoma and invasive ductal carcinoma. Breast Cancer Res Treat.

[16] Ohtani H, Sasano N (1980). Myofibroblasts and myoepithelial cells in human breast carcinoma. Virchows Arch A Pathol Anat Histol.

[17] Tsuchiya S, Li F (2005). Electron microscopic findings for diagnosis of breast lesions. Med Mol Morphol.

[18] Kalluri R, Zeisberg M (2006). Fibroblasts in cancer. Nat Rev Cancer.

[19] Franco OE, Shaw AK, Strand DW, Hayward SW (2010). Cancer associated fibroblasts in cancer pathogenesis. Semin Cell Dev Biol.

[20] Chauhan H (2003). There is more than one kind of myofibroblast: analysis of CD34 expression in benign, in situ, and invasive breast lesions. J Clin Pathol.

[21] Desmouliere A, Guyot C, Gabbiani G (2004). The stroma reaction myofibroblast: a key player in the control of tumor cell behavior. Int J Dev Biol.

[22] Mishra SK, Siddique HR, Saleem M (2012). S100A4 calcium-binding protein is key player in tumor progression and metastasis: preclinical and clinical evidence. Cancer Metastasis Rev.

[23] Kau S, Miller I, Tichy A, Gabriel C (2017). S100A4 (metastasin) positive mesenchymal canine mammary tumour spheroids reduce Tenascin C synthesis under DMSO exposure in vitro. Vet Comp Oncol.

[24] Yoshimura H, Otsuka A, Michishita M, Yamamoto M, Ashizawa M, Zushi M, Moriya M, Azakami D, Ochiai K, Matsuda Y, Ishiwata T, Kamiya S, Takahashi K (2019). Expression and roles of S100A4 in anaplastic cells of canine mammary carcinomas. Vet Pathol.

[25] LI Y, LIU S, LIU D, SUN S, KUANG C, DING Z, et al. Image scanning fluorescence emission difference microscopy based on a detector array. J Microsc 2017;266:288–297. doi:10.1111/jmi.12538, 3. 10.1111/jmi.12538 28199004

[26] Bashaw GJ (2010). Visualizing Axons in the Drosophila Central Nervous System Using Immunohistochemistry and Immunofluorescence. Cold Spring Harb Protoc.

[27] Gamba C d O, Damasceno KA, Ferreira IC, Rodrigues MA, Gomes DA, Alves MR (2019). The investigation of transcriptional repression mediated by ZEB2 in canine invasive micropapillary carcinoma in mammary gland. PLoS One.

[28] Kalluri R (2016). The biology and function of fibroblasts in cancer. Nat Rev Cancer.

[29] Grinnell F (1994). Fibroblasts, myofibroblasts, and wound contraction. J Cell Biol.

[30] Kuroda N, Sugimoto T, Takahashi T, Moriki T, Toi M, Miyazaki E, Hiroi M, Enzan H (2005). Invasive micropapillary carcinoma of the breast: an Immunohistochemical study of neoplastic and stromal cells. Int J Surg Pathol.

[31] Catteau X, Simon P, Noël J-C (2014). Myofibroblastic stromal reaction and lymph node status in invasive breast carcinoma: possible role of the TGF-β1/TGF-βR1 pathway. BMC Cancer.

[32] Gupta S, Hussain T, MacLennan GT, Fu P, Patel J, Mukhtar H (2003). Differential expression of S100A2 and S100A4 during progression of human prostate adenocarcinoma. J Clin Oncol.

[33] Zou M, Al-Baradie RS, Al-Hindi H, Farid NR, Shi Y (2005). S100A4 (Mts1) gene overexpression is associated with invasion and metastasis of papillary thyroid carcinoma. Br J Cancer.

[34] Ismail NI, Kaur G, Hashim H, Hassan MS (2008). S100A4 overexpression proves to be independent marker for breast cancer progression. Cancer Cell Int.

[35] Boye K, Mælandsmo GM (2010). S100A4 and metastasis. Am J Pathol.

[36] Fei F, Qu J, Zhang M, Li Y, Zhang S (2017). S100A4 in cancer progression and metastasis: A systematic review. Oncotarget.

[37] Baum J, Duffy HS (2011). Fibroblasts and Myofibroblasts: what are we talking about?. J Cardiovasc Pharmacol.

[38] Friedman G, Levi-Galibov O, David E, Bornstein C, Giladi A, Dadiani M, Mayo A, Halperin C, Pevsner-Fischer M, Lavon H, Mayer S, Nevo R, Stein Y, Balint-Lahat N, Barshack I, Ali HR, Caldas C, Nili-Gal-Yam E, Alon U, Amit I, Scherz-Shouval R (2020). Cancer-associated fibroblast compositions change with breast cancer progression linking the ratio of S100A4+ and PDPN+ CAFs to clinical outcome. Nat Can.

[39] Hasebe T, Iwasaki M, Akashi-Tanaka S, Hojo T, Shimizu C, Andoh M, Fujiwara Y, Shibata T, Sasajima Y, Kinoshita T, Tsuda H (2011). Atypical tumor-stromal fibroblasts in invasive ductal carcinomas of the breast treated with neoadjuvant therapy. Hum Pathol.

[40] Nurmik M, Ullmann P, Rodriguez F, Haan S, Letellier E (2020). In search of definitions: Cancer-associated fibroblasts and their markers. Int J Cancer.

[41] Fiori ME, Di Franco S, Villanova L, Bianca P, Stassi G, De Maria R (2019). Cancer-associated fibroblasts as abettors of tumor progression at the crossroads of EMT and therapy resistance. Mol Cancer.

[42] Eiro N, González L, Martínez-Ordoñez A, Fernandez-Garcia B, González LO, Cid S, Dominguez F, Perez-Fernandez R, Vizoso FJ (2018). Cancer-associated fibroblasts affect breast cancer cell gene expression, invasion and angiogenesis. Cell Oncol.

[43] Pettinato G, Manivel CJ, Panico L, Sparano L, Petrella G (2004). Invasive micropapillary carcinoma of the breast : Clinicopathologic study of 62 cases of a poorly recognized variant with highly aggressive behavior. Am J Clin Pathol.

[44] Tavassoli F, 2nd (1999). Papillary lesions. Pathology of the breast.

[45] Hill CB, Yeh I-T (2005). Myoepithelial cell staining patterns of papillary breast lesions. Am J Clin Pathol.

[46] Russell TD, Jindal S, Agunbiade S, Gao D, Troxell M, Borges VF, Schedin P (2015). Myoepithelial cell differentiation markers in ductal carcinoma in situ progression. Am J Pathol.

[47] Lo P-K, Zhang Y, Yao Y, Wolfson B, Yu J, Han S-Y, Duru N, Zhou Q (2017). Tumor-associated myoepithelial cells promote the invasive progression of ductal carcinoma in situ through activation of TGFβ signaling. J Biol Chem.

[48] Gama A, Alves A, Gartner F, Schmitt F (2003). p63: a novel Myoepithelial cell marker in canine mammary tissues. Vet Pathol.

[49] Yoshimura H, Nakahira R, Kishimoto TE, Michishita M, Ohkusu-Tsukada K, Takahashi K (2014). Differences in indicators of malignancy between luminal epithelial cell type and Myoepithelial cell type of simple solid carcinoma in the canine mammary gland. Vet Pathol.

[50] Barbareschi M, Pecciarini L, Cangi MG, Macrì E, Rizzo A, Viale G, Doglioni C (2001). p63, a p53 homologue, is a selective nuclear marker of Myoepithelial cells of the human breast. Am J Surg Pathol.

[51] Ding L, Su Y, Fassl A, Hinohara K, Qiu X, Harper NW, Huh SJ, Bloushtain-Qimron N, Jovanović B, Ekram M, Zi X, Hines WC, Alečković M, Gil del Alcazar C, Caulfield RJ, Bonal DM, Nguyen QD, Merino VF, Choudhury S, Ethington G, Panos L, Grant M, Herlihy W, Au A, Rosson GD, Argani P, Richardson AL, Dillon D, Allred DC, Babski K, Kim EMH, McDonnell CH, Wagner J, Rowberry R, Bobolis K, Kleer CG, Hwang ES, Blum JL, Cristea S, Sicinski P, Fan R, Long HW, Sukumar S, Park SY, Garber JE, Bissell M, Yao J, Polyak K (2019). Perturbed myoepithelial cell differentiation in BRCA mutation carriers and in ductal carcinoma in situ. Nat Commun.

[52] Winterford CM, Walsh MD, Leggett BA, Jass JR (1999). Ultrastructural localization of epithelial Mucin Core proteins in colorectal tissues. J Histochem Cytochem.

[53] Wei J, Cui L, Liu F, Yu F, Lang R, Feng G (2010). E-selectin and Sialyl Lewis X expression is associated with lymph node metastasis of invasive micropapillary carcinoma of the breast. Int J Surg Pathol.

[54] Cassali G, Jark P, Gamba C, Damasceno K, Estrela-Lima A, Nardi A (2020). Consensus Regarding the Diagnosis, Prognosis and Treatment of Canine and Feline Mammary Tumors - 2019. Brazilian J Vet Pathol.

[55] Elston C (1991). W. EIO. Pathological prognostic factors in breast cancer. I. the value of histological grade in breast cancer: experience from a large study with long-term follow-up. Histopathology..

[56] EIO ECW. Assessment of histological grade. In: Systemic Pathology—The Breast. Third: Elsevier Science Health Science Division; 1998. p. 365–84.

[57] Nunes FC, Campos CB, Teixeira SV, Bertagnolli AC, Lavalle GE, Cassali GD (2018). Epidemiological, clinical and pathological evaluation of overall survival in canines with mammary neoplasms. Arq Bras Med Veterinária e Zootec.

[58] Faria JAQA, de Andrade C, Goes AM, Rodrigues MA, Gomes DA (2016). Effects of different ligands on epidermal growth factor receptor (EGFR) nuclear translocation. Biochem Biophys Res Commun.

[59] Rodrigues MA, Gamba CO, Faria JAQA, Ferreira Ê, Goes AM, Gomes DA, Cassali GD (2016). Inner nuclear membrane localization of epidermal growth factor receptor (EGFR) in spontaneous canine model of invasive micropapillary carcinoma of the mammary gland. Pathol Res Pract.

[60] de Miranda MC, Rodrigues MA, de Angelis Campos AC, Faria JAQA, Kunrath-Lima M, Mignery GA, Schechtman D, Goes AM, Nathanson MH, Gomes DA (2019). Epidermal growth factor (EGF) triggers nuclear calcium signaling through the intranuclear phospholipase Cδ-4 (PLCδ4). J Biol Chem.

[61] Bertagnolli AC, Cassali GD, Genelhu MCLS, Costa FA, Oliveira JFC, Gonçalves PBD (2009). Immunohistochemical expression of p63 and ΔNp63 in mixed tumors of canine mammary glands and its relation with p53 expression. Vet Pathol.

[62] de Oliveira JT, Pinho SS, de Matos AJ, Hespanhol V, Reis CA, Gärtner F (2009). MUC1 expression in canine malignant mammary tumours and relationship to clinicopathological features. Vet J.

[63] Damasceno KA, Bertagnolli AC, Estrela-Lima A, Ribeiro LG, Rabelo BS, Campos CB (2012). Versican expression in canine carcinomas in benign mixed tumours: is there an association with clinical pathological factors, invasion and overall survival?. BMC Vet Res.

[64] Dolka I, Sapierzyński R, Król M (2013). Retrospective study and immunohistochemical analysis of canine mammary sarcomas. BMC Vet Res.

[65] Sachdeva M, Mo Y-Y (2010). MicroRNA-145 suppresses cell invasion and metastasis by directly targeting Mucin 1. Cancer Res.

[66] Graubner FR, Boos A, Aslan S, Kücükaslan I, Kowalewski MP (2018). Uterine and placental distribution of selected extracellular matrix (ECM) components in the dog. Reproduction..

[67] Monteiro LN, Rodrigues MA, Gomes DA, Salgado BS, Cassali GD (2018). Tumour-associated macrophages: relation with progression and invasiveness, and assessment of M1/M2 macrophages in canine mammary tumours. Vet J.

[68] Heilemann M (2010). Fluorescence microscopy beyond the diffraction limit. J Biotechnol.

