# Supplementary material for: Characterization of neoplastic cells outlining the cystic space of invasive micropapillary carcinoma of the canine mammary gland
Source: BMC Vet Res. 2021 Mar 24;17:130. doi: 10.1186/s12917-021-02807-y (PMC7992814; doi:10.1186/s12917-021-02807-y)
Supplement: Supplementary file 6 — Additional file 6: Table 1 [file 12917_2021_2807_MOESM6_ESM.docx]

**Supplementary Table 1: Negative and positive staining summary by IHC and IF in**

**IMPC of the canine mammary gland.**

| **Staining** | **IHC** | **IF** |
| --- | --- | --- |
| S100A4 | positive | positive |
| αSMA | positive | positive |
| Vimentin | positive | positive |
| MUC1 | positive | positive |
| p63 | negative | negative |
| CD31 | negative | negative |
| Von Willebrand Factor | negative | negative |
| Cytokeratin | positive | positive |
